# Systematic review of methods for quantifying teamwork in the operating theatre

**DOI:** 10.1002/bjs5.40

**Published:** 2018-02-15

**Authors:** N. Li, D. Marshall, M. Sykes, P. McCulloch, J. Shalhoub, M. Maruthappu

**Affiliations:** ^1^ Department of General Surgery Wexham Park Hospital Slough UK; ^2^ Department of Medicine Imperial College London London UK; ^3^ Department of Surgery and Cancer Imperial College London London UK; ^4^ Nuffield Department of Surgery University of Oxford Oxford UK

## Abstract

**Background:**

Teamwork in the operating theatre is becoming increasingly recognized as a major factor in clinical outcomes. Many tools have been developed to measure teamwork. Most fall into two categories: self‐assessment by theatre staff and assessment by observers. A critical and comparative analysis of the validity and reliability of these tools is lacking.

**Methods:**

MEDLINE and Embase databases were searched following PRISMA guidelines. Content validity was assessed using measurements of inter‐rater agreement, predictive validity and multisite reliability, and interobserver reliability using statistical measures of inter‐rater agreement and reliability. Quantitative meta‐analysis was deemed unsuitable.

**Results:**

Forty‐eight articles were selected for final inclusion; self‐assessment tools were used in 18 and observational tools in 28, and there were two qualitative studies. Self‐assessment of teamwork by profession varied with the profession of the assessor. The most robust self‐assessment tool was the Safety Attitudes Questionnaire (SAQ), although this failed to demonstrate multisite reliability. The most robust observational tool was the Non‐Technical Skills (NOTECHS) system, which demonstrated both test–retest reliability (P > 0·09) and interobserver reliability (Rwg = 0·96).

**Conclusion:**

Self‐assessment of teamwork by the theatre team was influenced by professional differences. Observational tools, when used by trained observers, circumvented this.

## Introduction

The past decade has seen a dramatic shift in understanding of surgical performance and outcomes. In addition to surgeons' technical proficiency, non‐technical skills have been implicated in clinical outcomes after surgery and operating theatre efficiency. These non‐technical skills include, in addition to teamwork, attitudes towards safety, situational awareness, decision‐making, communication and theatre environment^1^, ^2^, ^3^, ^4^, ^5^, ^6^, ^7^, ^8^, ^9^, ^10^. This review was designed to focus on teamwork. Therefore, tools that did not explicitly claim to involve teamwork metrics in their measurement were not considered.

A variety of tools with varying degrees of validity and reliability exist. They fall broadly into two categories: self‐assessment by operating theatre staff and direct observation of the theatre team by others. Without a widely accepted method of quantifying teamwork within the operating theatre, it is difficult to evaluate teamwork in a consistent and comparable manner.

A number of problems exist when attempting to quantify teamwork. A comprehensive definition has not been agreed, reflecting the variations in content and approach to measuring teamwork. Pragmatic factors such as cost and practicality may influence whether one tool is selected over another for clinical purposes. However, selected tools should be valid and reliable. Theoretically, comprehensive tools are not useful scientifically if invalid or unreliable when tested in unsimulated environments; nor can validity or reliability be sacrificed for ease of implementation and cost. Although previous authors^11^ ^12^ have commented on the validity and reliability of teamwork tools, none has focused specifically on teamwork in the operating theatre. This is an important distinction to make, as many authors would agree that teamwork measures a set of processes that are specific to a situation. To align with this definition, this study presents a more targeted and focused approach by excluding studies that relate to, for example, simulated settings or military trauma.

## Methods

### Search strategy

**Figure 1:**
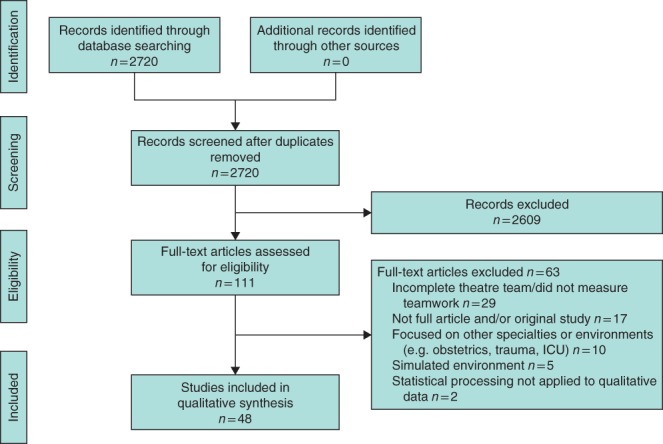
Flow diagram showing selection of articles for review

The search strategy was completed according to the PRISMA recommendations for systematic reviews^13^ (*Fig*. 1). The Ovid search engine was used to interrogate the MEDLINE and Embase databases using the following individual search strategies. MEDLINE: (*patient care team/ or teamwork.mp. or cumulative experience.mp.) and (surg*.mp. or operation op * operating rooms/ma) and (quality indicators, health care/ or complications.mp. or outcomes.mp. or safety.mp. or performance.mp. or mortality.mp.). EMBASE: (teamwork/ or cumulative experience.mp.) and (surg*.mp. or operating room/ or surgery/ or operation.mp.) and (health care quality/ or complications.mp. or safety.mp. or outcomes.mp. or performance.mp. or mortality.mp.). The reference lists of included articles were searched for additional studies. Two independent reviewers assessed the titles and abstracts of all identified articles to determine eligibility. Eligible studies were assessed in full with a third reviewer if information retrieved from the titles and abstracts was insufficient to determine inclusion. A fourth independent reviewer was responsible for resolving any dispute in initial study inclusion/exclusion.

### Study selection

The papers were selected for review based on the following inclusion criteria: original paper; English version obtainable; focuses on measurement of teamwork as defined by the authors themselves; includes statistical processing of data related to measurement of teamwork (for quantitative studies); and investigates operating theatre teams. The following exclusion criteria were applied: abstract only; no statistical processing of data related to measurement of teamwork (for quantitative studies); teamwork not assessed holistically (for example, choosing to investigate communication only); and involves teamwork outside the operating theatre. Authors independently reviewed articles and all queries were resolved.

### Data of interest

Data that were extracted and synthesized for analysis included: first author, aim of the study, study design, country of origin, setting and specialty, use of crew resource management, number of teams, size of teams, number of surgical procedures, teamwork intervention used, duration/frequency of intervention, number of surgeons, experience of surgical team, outcome measures (mortality, morbidity, team efficiency, duration of operation, ‘never’ events, team opinions, teamwork quality), and feedback provision. All included articles were read in full to evaluate the methods used by authors to show content validity, predictive validity, reliability between test sites, and reliability between observers for observational tools. Only sections of tools relating to teamwork, as defined by the creators of each tool, were analysed. Other fields that may comprise part of a broader tool, such as the job satisfaction domain of the Safety Attitudes Questionnaire (SAQ), were not taken into account.

### Analysis

Study characteristics and outcomes were summarized and contrasted using descriptive methods. Critical assessments of content validity, predictive validity and concurrent validity were made. Although largely subjective^14^, content validity was deemed to be of greater value in tools that had shown high internal agreement or evidence of translation from other fields as opposed to simple transposition. Predictive validity was judged by the impact of training on teamwork scores, that is whether one can predict whether staff had undergone team training from scores registered before and after intervention. Concurrent validity is displayed with statistical correlation with other factors thought to be related to teamwork. Tools were also deemed to be more valid if multiple facets of validity were displayed. Statistical measures of inter‐rater agreement (Rwg and Cohen's κ) and inter‐rater reliability (intraclass coefficient, ICC) were also compared. Non‐significant scores across time intervals or institutions were taken as markers of test–retest reliability**.** Heterogeneity in study design and variation in outcome, population and setting precluded meta‐analysis. Therefore, a predominantly qualitative approach was adopted.

## Results

Of 2720 citations, 48 articles were included for review. Studies were published between 2002 and 2015, encompassing 59 306 patients and 13 453 staff at 228 sites. These articles comprised 24 cross‐sectional studies, 21 prospective studies, one retrospective and two qualitative studies (*Tables* 1 and 2).

**Table 1:** Teamwork measurement tools using self‐assessment

Tool	Design	Content validity	Predictive validity	Concurrent validity	Test–retest reliability
Teamwork climate of SAQ^2^ ^3^, ^15^, ^16^, ^17^, ^18^, ^19^, ^20^, ^21^	Likert scale survey	Developed from FMAQ, used in aviation. Psychometric basis, minimal alterations Cronbach's α = 0·78 for a sample of items on the SAQ teamwork scale (same profession, same site)^3^	Scores were better in the site that had received teamwork training compared with one that had not^2^	Correlation with theatre efficiency^17^	No, two sites had a significantly different baseline score^2^
TeamSTEPPS questionnaire^4^	Likert scale survey	As part of government‐ sponsored TeamSTEPPS programme	Scores improved after TeamSTEPPS training	No	n.r.
MTTQ^22^	Likert scale survey	Statistical method of factor analysis	No	No	No, different sites had significantly different MTTQ responses (*P* < 0·001)
ORMAQ^23^	Likert scale survey	Adapted from aviation and other languages by 3 surgeons	No	No	n.r.
Study‐specific survey^1^	Likert scale survey	Claims validated, unable to find method of validation	Teamwork scores of surgeons and anaesthetists improved after team training; those of nurses did not	No	n.r.
Study‐specific survey^5^	Yes/no responses	No	Increased perceptions of teamwork after training	No	n.r.
Study‐specific survey^9^	Two parts: yes/no and Likert scale survey	Based on literature review	After safety checklist implementation, greater proportion of surgeons reported positive teamwork events	No	n.r.
Study‐specific survey^24^	Likert scale survey	Input from orthopaedic surgeons, anaesthetists, ICU and physicians	Improved perceptions of teamwork after perioperative checklist implementation	No	n.r.
Study‐specific survey^25^	Free‐text answers calculated into score out of 5	No	No	No	n.r.
Study‐specific survey^26^	Self reporting of statements taken from observational tools	Survey items translated from observational tools (NOTSS and ANTS)	No	No	n.r.

SAQ, Safety Attitudes Questionnaire; FMAQ, Flight Management Attitudes Questionnaire; TeamSTEPPS, Team Strategies and Tools to Enhance Performance and Patient Safety; n.r., not reported; MTTQ, Medical Team Training Questionnaire; ORMAQ, Operating Room Management Attitudes Questionnaire; NOTSS, Non‐technical Skills for Surgeons; ANTS, Anaesthetists' Non‐Technical Skills.

**Table 2:** Teamwork measurement using direct observation

Tool	Design	Content validity	Predictive validity	Concurrent validity	Test–retest reliability	Inter‐rater reliability (ICC) and agreement (κ, Rwg)
NOTECHS^27^, ^28^, ^29^, ^30^, ^31^, ^32^, ^33^	Scale: observed behaviours	Translated from aviation by theatre experts and human factors experts^27^	Improved scores after team training (*P =* 0·005)^27^ Improved scores after team and systems training (*P =* 0·025)^33^	Expected and observed correlation with glitch rate (*P =* 0·045)^28^	0·09 < *P* < 0·64 across 5 sites (non‐significant variation)^28^ Non‐significant variation across different time intervals^27^	Rwg = 0·96^27^ ICC = 0·73–0·88^28^
OTAS^10^ ^34^, ^35^, ^36^, ^37^, ^38^, ^39^, ^40^	Checklist: tasks and scale: observed behaviours	Theatre and human factors experts involved in development	No	Adverse correlation between impact of distractions and completion of patient‐related tasks (*P* < 0·050)^6^ ^10^	n.r.	Cohen's κ > 0·40^34^ Pearson's coefficient = 0·71^38^ ICC = 0·42–0·90^40^. In German operating theatres: κ > 0·40 in 70% of scale items, ICC = 0·78–0·89^39^
SO‐DIC‐OR^41^	Checklist: observed behaviours	Representative sample of theatre team involved in development	No	No	n.r.	Cohen's κ = 0·74–0·95 including for ‘tired’ observers
Coding of field notes^42^	Scale: impact of coded field notes	No	No	No	n.r.	No, each observer had a different role
Mayo‐HPTS^43^ ^44^	Checklist: tasks and scale: behaviours	Validated for crew resource management^44^	Improved scores after team training (*P =* 0·01)	No	n.r.	Cohen's κ = 0·46–0·97^43^
METEOR^45^	Checklist: tasks	Scale items verified by agreement between theatre experts	No	No	n.r.	Observers ‘calibrated’ until Cohen's κ > 0·70 Observer agreement for cases n.r.
NOTSS^40^ ^46^	Scale: behaviours	Theatre experts involved in development	No	Good correlation with Cannon‐Bowers scale^32^	n.r.	ICC = 0·12–0·83^47^
Cannon‐Bowers^46^ ^48^	Literature review	Based on psychological theory	No	Good correlation with NOTSS	n.r.	Cronbach's α = 0·80
HFRS‐M^47,49^	Scale: behaviours	Took elements of LOSA checklist for aviation	Briefing workshops and simulation had no significant effect on scores	No	n.r.	Cronbach's α = 0·89^47^
Study‐specific survey^7^	Scale: observed behaviours	Based on behavioural markers	No	No	n.r.	Observers ‘calibrated’ Rwg = 0·85 after training. Observer agreement for cases n.r.
Study‐specific survey^50^	Checklist: coded events	Based on previously validated tool for assessing mental fitness and concerns	No	No	n.r.	Cohen's κ = 0·77

ICC, intraclass coefficient; NOTECHS, Non‐Technical Skills; OTAS, Observational Teamwork Assessment for Surgery; SO‐DIC‐OR, Simultaneous Observation of Distractions and Communication in the Operating Room; Mayo‐HPTS, Mayo High Performance Teamwork Score; METEOR, Metric for Evaluating Task Execution in the Operating Room; NOTSS, Non‐technical Skills for Surgeons; HFRS‐M, Modified Human Factors Rating Scale; LOSA, Line Oriented Safety Audit.

### Self‐assessment methods

Self‐assessment tools were used in 18 studies across 194 sites (*Table* 1). The most popular tool was the teamwork subsection or ‘climate’ of the SAQ^2^ ^3^, ^15^, ^16^, ^17^, ^18^, ^19^, ^20^.

#### *Content validity*

A number of tools contained evidence of content validity, although the SAQ was the only one that demonstrated high internal agreement by users (Cronbach's α = 0·78)^3^. The SAQ also had the benefit of translation from a well validated tool used in aviation, a feature shared with the Operating Room Management Attitudes Questionnaire (ORMAQ). However, adaptations to the operating theatre were largely semantic^11^ ^16^, ^51^. Tools had also been borrowed from other medical specialties including the TeamSTEPPS training^4^, medical team training^22^, and ICU and trauma^24^, although none exhibited convincing adaptation to the operating room specifically. Some studies did not demonstrate content validity^5^ ^25^.

#### *Predictive validity*

Although statistically significant improvements in SAQ scores were demonstrated after teamwork training^2^, this finding was not reproduced in all studies^18^ ^20^, ^21^. Other tools showed improvement in teamwork scores after training and implementation of a surgical safety checklist^4^ ^5^, ^9^ ^24^, although the improvements were not always seen in representatives of the nursing profession^1^.

#### *Concurrent validity*

SAQ scores correlated with theatre efficiency, but not with an independent scoring system for communication^3^ ^17^.

#### *Reliability*

The SAQ did not appear reliable in retest conditions, with significant differences in scores across institutions and across time intervals without intervention^2^. Similarly, the Medical Team Training Questionnaire (MTTQ) also did not display test–retest reliability across different institutions^22^.

A number of studies^1^ ^5^, ^15^ ^16^, ^19^ ^22^, ^23^ showed that perceptions of teamwork varied between the professions that constitute the operating team. For example, surgeons rated the teamwork of their theatre colleagues higher than that of anaesthetists or nurses^15^. This finding was present regardless of the assessment method. Furthermore, members of each profession tended to give the highest ratings of teamwork to their own profession^11^ ^15^. All forms of self‐assessed scores for teamwork included some form of questionnaire or survey, many of which were based on a Likert scale. The response rate to these surveys varied from 45 to 87 per cent (*Table* 3). For studies using the SAQ, mean response rates varied from 52 to 87 per cent.

**Table 3:** Reported response rates for self‐assessment of teamwork

Reference	Mean survey response rate (%)
Papaconstantinou *et al*.^9^	45
Flin *et al*.^23^	48
Davenport *et al*.^3^	52
Bleakley *et al*.^2^	68
Sexton *et al*.^16^	71
Makary *et al*.^15^	77
Mills *et al*.^22^	80
Kawano *et al*.^21^	87

### Methods of direct observation

Twenty‐eight studies quantified teamwork using direct observation (*Table* 2). The two most commonly used tools were the Observational Teamwork Assessment for Surgery (OTAS)^34^, ^35^, ^36^, ^37^, ^38^, ^39^ ^52^ and the Non‐Technical Skills (NOTECHS) system^27^, ^28^, ^29^, ^30^, ^31^, ^32^, ^33^.

#### *Content validity*

NOTECHS benefited from development from a previously well validated tool used in aviation^27^, whereas another method was developed from a tool for assessing mental fitness^50^. The majority of the observational tools had been developed using theatre experts, or adapted from existing tools by theatre experts. Exceptions include the Mayo High Performance Teamwork Score (HPTS) and the Modified Human Factors Rating Scale (HFRS‐M), which comprised elements taken directly from crew resource management without translation^44^ ^47^, ^49^, and the Cannon‐Bowers scale based on psychological theory^46^ ^48^. NOTECHS has also been validated in vascular, orthopaedic and general surgery^27^ ^28^, ^31^. OTAS also shows evidence of validation in multiple specialties, having been tested in urology, vascular and general surgery, and in operating theatres in Germany^34^ ^36^, ^39^.

#### *Predictive validity*

NOTECHS consistently demonstrated highly significant improvement in teamwork scores after teamwork training^27^ ^33^. The only other observational tool to demonstrate predictive validity was the Mayo‐HPTS, which also showed statistically significant improvements after team training^44^. Team training and simulation did not have any significant effect on HFRS‐M scores^49^.

#### *Concurrent validity*

NOTECHS scores correlated inversely with ‘glitch rate’, whereas OTAS scores inversely correlated with the impact of distractions^6^ ^10^. NOTSS and the Cannon‐Bowers scale correlated well with each other^40^ ^46^, ^48^.

#### *Test–retest reliability*

NOTECHS was the only tool to demonstrate reliability when tested across different sites and different time intervals^27^ ^28^.

#### *Inter‐rater reliability and agreement*

NOTECHS showed superior statistical measures of inter‐rater reliability (ICC = 0·73–0·88)^28^, with relatively small ranges in the statistical measures of inter‐rater reliability, compared with OTAS (ICC = 0·42–0·90)^40^ and NOTSS (ICC = 0·12–0·83)^47^. Inter‐rater agreement was strong for NOTECHS (Rwg = 0·96)^27^, Simultaneous Observation of Distractions and Communication in the Operating Room (SO‐DIC‐OR) (κ = 0·74–0·90) and a study‐specific survey (κ = 0·77)^50^, but less strong for OTAS (κ > 0·40) and Mayo‐HPTS (κ > 0·46).

### Qualitative studies

Two studies used structured interviews with a combined total of seven surgeons, 25 nurses and eight anaesthetists. One study produced ethnographic field notes on 35 procedures. ‘Differences in professional culture’ between surgeons, anaesthetists and nurses was identified as a major influence in team communication^53^. Operating theatre staff also implicated the ‘role of the institution’ in teamwork and communication. Perceived barriers to effective teamwork included a lack of ‘open communication’ and ‘dominance and hierarchy’^54^. Field notes of observed communication exchanges in the operating theatre showed themes such as ‘mimicry’ (for example, junior surgeons mimicking the behaviours of fellows and consultant), ‘withdrawal’ (typically juniors withdrawing from tense communication between other team members), and ‘association’ (attitudes towards a certain individual being extended to members of their professional subteam)^54^.

## Discussion

As far as validity and reliability were concerned, NOTECHS was the most valid and reliable observational tool for measuring teamwork. The NOTECHS score also demonstrated predictive validity, concurrent validity, superior test–retest reliability and superior inter‐rater reliability^28^. NOTECHS has been used across a range of specialties including general, vascular and orthopaedic surgery^27^ ^28^, ^31^. It was adapted from a synonymous, well accepted score used in aviation, which has roots in psychological theory^55^. The changes between the aviation NOTECHS and the operating theatre NOTECHS involved the input of surgical, anaesthetic and nursing experts^27^.

OTAS has been validated in urology, vascular surgery and general surgery^36^. Its content, like that of NOTECHS, has contributions from psychological and clinical expertise. Despite this, a proportion of OTAS components (behaviours or tasks) were consistently not witnessed in practice^12^ ^36^, ^37^. After translation to German operating theatres, inter‐rater agreement also remained poor (κ < 0·40 in 30 per cent of tool items)^39^. This may be explained by suboptimal team performance, but also casts doubt on its content validity and tool reliability. There was no evidence for the predictive validity of OTAS, and no evidence of test–retest reliability.

Several important limitations of self‐reported tools have been identified. It is difficult to obtain a meaningful score for the whole team. Studies consistently showed that assessment of the teamwork of colleagues, and of the whole team, was different for each profession^1^ ^5^, ^11^ ^15^, ^16^ ^22^. Participants tended to rate their own specialty the highest on scales of communication and teamwork. Assuming honest ratings not coloured by factionism, this suggests that each profession has different ideas of what comprises good teamwork. Qualitative studies have identified ‘differences in professional culture’ as a major influence on teamwork^53^. The frequent occurrence of behaviours such as ‘mimicry’ and ‘association’ substantiate this. Junior staff belonging to a specialty often mimic the negative teamwork behaviours of their seniors, and members of other specialties associate juniors with negative traits of seniors^54^. It appears challenging for individuals in theatre subteams adequately to assess themselves and their colleagues from other professions.

Self‐assessed methods of teamwork appear to be greatly influenced by the site at which the work was done. Two studies^2^ ^22^ showed significantly different scores at different sites, and no other studies reported on this subject. This may be an example of failure to show test–retest reliability. Otherwise, if the difference in perceived teamwork between sites was true, it can be better described by the difference in the pattern of responses, not the absolute score. In this case, self‐assessment is suitable for qualitative investigation of interactions between team members, but not useful as an overall quantifier of teamwork. Either self‐assessment tools are unreliable, or they are more useful in qualitative assessment.

The relative abundance of operating room nurses and scarcity of anaesthetists presents a further problem for self‐assessment of teamwork. Of the studies included, the combined ratio of nurses to surgeons to anaesthetists was roughly 3 : 2 : 1 (*Table* S1, supporting information). Consequently, a simple arithmetic combination of scores from each profession would over‐represent nursing perspectives and under‐represent anaesthetic perspectives. Problems with sampling were also evident, as shown by the wide range of response rates between studies, and between sites within a study. The lack of sampling methods could allow studies to have an inherent bias, self‐selecting for individuals with an interest in teamwork.

A valid tool measures accurately and precisely what it is designed to measure in the real world. Broadly, there are three types of validity relevant to this review: content validity, predictive validity and concurrent validity. A tool is deemed to have content validity if it actually measures what it was intended to measure in a given content. This remains largely a qualitative judgement despite attempts to quantify it^14^. Many authors have attempted to show content validity by involving psychological experts and operating theatre experts.

In the traditional sense, a tool has predictive validity if it can be used to make reasonable predictions based on what it measures. However, teamwork in the operating theatre is not proven to have causal relationships with other measurable variables. One must first establish causation between teamwork and another variable before going back to ascertain whether a tool that measures teamwork also has predictive validity for that variable. At this stage, true predictive validity for teamwork relating to other variables cannot be demonstrated. However, by considering scores before and after training, the presence or absence of training may be inferred if a tool shows predictive validity. Concurrent validity is similar to predictive validity, but the variable that is correlated to teamwork is happening at the same time.

Any tool deemed to be reliable must show test–retest reliability. As such, scores should not be affected by testing at different sites or in different time intervals without intervention. In addition, observational tools must show reliability between raters/observers. This is different from inter‐rater agreement. Raters can agree exactly on a test, but unreliably so; likewise, raters may reliably disagree over their observations. The studies employed a variety of statistical tools to examine these issues (*Table* 2). Rwg and Cohen's κ are measures of inter‐rater agreement; ICC values provide an estimate of reliability between raters.

Some studies focused on a single‐specialty approach to validity, perhaps on the premise that teamwork was not only situation‐dependent (operating theatre as opposed to emergency teams), but also task‐dependent. There was no evidence that requirements for teamwork varied by surgical specialty. As OTAS and NOTECHS have been validated in multiple specialties, there is evidence to the contrary^27^ ^29^, ^30^ ^35^.

A common shortcoming was that some tools that have been validated in other settings were directly transferred to the operating theatre environment without adaptation or validity testing. Common settings included: crew resource management^43^ ^47^, ^49^, medical as opposed to surgical teams^4^ ^22^, ICU and trauma^24^. Some authors^1^ ^5^, ^9^ ^25^, ^42^ used study‐specific tools without reporting processes of development and validation.

Furthermore, statistical tests must be applied appropriately. For example, Pearson's coefficient, although used by authors^38^ for quantifying correlation between raters for teamwork, is a tool for estimating correlations between variables that do not share a metric and variance, and, therefore, inappropriate for use to correlate observations of two raters on the same score^56^ ^57^.

Meta‐analysis was not attempted and heterogeneity of the different tools limits the conclusions of this review. Within these limitations, it seems that the ideal tool should employ trained observers, must be valid for the operating theatre and reliable between observers, specialties and sites. So far, the tool closest to fulfilling these criteria is the NOTECHS. Future research might aim to demonstrate its reliability for longer procedures, similar to the SO‐DIC‐OR.

## Supporting information


**Table S1** Self‐assessment responses by staff profession
