# Supplementary material for: Systematic review of methods for quantifying teamwork in the operating theatre
Source: BJS Open. 2018 Feb 15;2(2):42–51. doi: 10.1002/bjs5.40 (PMC5952378; doi:10.1002/bjs5.40)
Supplement: Supplementary file 1 — Table S1 Self‐assessment responses by staff profession [file BJS5-2-42-s001.docx]

**BJS5_40**

**Systematic review of** **methods for quantifying teamwork in the operating theatre**

N. Li, D. Marshall, M. Sykes, P. McCulloch, J. Shalhoub and M. Maruthappu

**Table S1** Self-assessment responses by staff profession

| **First Author** | **Year** | **Design** | **No. of sites** | **No. of nurses** | **% nurses** | **No. of anaesthetists** | **% anaesthetists** | **No. of surgeons** | **% surgeons** | **Total no. of respondants** |
| --- | --- | --- | --- | --- | --- | --- | --- | --- | --- | --- |
| Wauben | 2011 | cross-sectional survey | 5 | 97 | 41 | 18 | 8 | 66 | 28 | 235 |
| Kawano | 2014 | prospective | 1 | 110 | 68 | 18 | 11 | 34 | 21 | 162 |
| Mills | 2008 | Cross-sectional survey | 6 | 139 | 60 | 49 | 21 | 45 | 19 | 233 |
| Haynes | 2011 | prospective | 8 | 84 | 16 | 63 | 12 | 82 | 15 | 538 |
| Papaconstantinou | 2013 | Prospective | 1 | 153 | 35 | 104 | 24 | 180 | 41 | 437 |
| Makary | 2006 | cross-sectional survey | 60 | 1058 | 50 | 170 | 8 | 222 | 10 | 2135 |
| Paige | 2009 | prospective | 1 | 5 | 50 | - | - | 1 | 10 | 10 |
| Flin | 2006 | Cross-sectional survey | 17 | 231 | 66 | - | - | 121 | 34 | 352 |
| Carney | 2010 | cross-sectional survey | 34 | 378 | 24 | - | - | 312 | 20 | 1581 |
| Davenport | 2007 | cross-sectional survey | 52 | 3432 | 56 | - | - | 1881 | 31 | 6084 |
| Halverson | 2009 | Prospective | 1 | - | - | - | - | - | - | 1150 |
| Forse | 2011 | Prospective | 1 | - | - | - | - | - | - | - |
| Awad | 2005 | Prospective | 1 | - | - | - | - | - | - | - |
| Bleakley | 2006 | Prospective | 1 | - | - | - | - | - | - | 221 |
| Sexton | 2006 | Cross-sectional survey | same sample as in Makary et al. |  |  |  |  |  |  |  |
| Stepaniak | 2012 | cross-sectional survey | 2 | - | - | - | - | - | - | - |
| Wolf | 2010 | prospective | 1 | - | - | - | - | - | - |  |
| Berenholtz | 2009 | prospective | 1 | - | - | - | - | - | - | 40 |
| Bohmer | 2012 | prospective | 1 | - | - | - | - | - | - | 71 |
|  |  |  |  |  |  |  |  |  |  |  |
| **Sum** |  |  | 194 |  |  |  |  |  |  | 13249 |
| **Mean** |  |  |  |  | 46 |  | 14 |  | 23 |  |
| **Standard deviation** |  |  |  |  | 17.44923821 |  | 6.864224682 |  | 10.35537045 |  |
